# Non-invasive Assessment of Fecal Stress Biomarkers in Hunting Dogs During Exercise and at Rest

**DOI:** 10.3389/fvets.2020.00126

**Published:** 2020-04-21

**Authors:** Augusta Zannoni, Marco Pietra, Alba Gaspardo, Pier Attilio Accorsi, Monica Barone, Silvia Turroni, Luca Laghi, Chenglin Zhu, Patrizia Brigidi, Monica Forni

**Affiliations:** ^1^Department of Veterinary Medical Sciences, University of Bologna, Bologna, Italy; ^2^Health Sciences and Technologies—Interdepartmental Center for Industrial Research (CIRI-SDV), Alma Mater Studiorum—University of Bologna, Bologna, Italy; ^3^Unit of Microbial Ecology of Health, Department of Pharmacy and Biotechnology, University of Bologna, Bologna, Italy; ^4^Interdepartmental Centre for Agri-Food Industrial Research, University of Bologna, Bologna, Italy; ^5^Department of Agro-Food Science and Technology, Centre of Foodomics, University of Bologna, Cesena, Italy

**Keywords:** dog, exercise, stress markers, stool, welfare

## Abstract

Intense exercise causes to organisms to have oxidative stress and inflammation at the gastrointestinal (GI) level. The reduction in intestinal blood flow and the exercise-linked thermal damage to the intestinal mucosa can cause intestinal barrier disruption, followed by an inflammatory response. Furthermore, the adaptation to exercise may affect the gut microbiota and the metabolome of the biofluids. The aim of the present research was to evaluate the presence of a GI derangement in hunting dogs through a non-invasive sampling as a consequence of a period of intense exercise in comparison with samples collected at rest. The study included nine dogs that underwent the same training regime for hunting wild boar. In order to counterbalance physiological variations, multiple-day replicates were collected and pooled at each experimental point for each dog. The samples were collected immediately at rest before the training (T0), after 60 days of training (T1), after 60 days of hunting wild boar (T2), and finally, at 60 days of rest after hunting (T3). A number of potential stress markers were evaluated: fecal cortisol metabolites (FCMs) as a major indicator of altered physiological states, immunoglobulin A (IgA) as an indicator of intestinal immune protection, and total antioxidant activity [total antioxidant capacity (TAC)]. Since stool samples contain exfoliated cells, we investigated also the presence of some transcripts involved in GI permeability [occludin (OCLN), protease-activated receptor-2 (PAR-2)] and in the inflammatory mechanism [interleukin (IL)-8, IL-6, IL-1b, tumor necrosis factor alpha (TNFα), calprotectin (CALP), heme oxygenase-1 (HO-1)]. Finally, the metabolome and the microbiota profiles were analyzed. No variation in FCM and IgA content and no differences in OCLN and CALP gene expression between rest and training were observed. On the contrary, an increase in PAR-2 and HO-1 transcripts, a reduction in total antioxidant activity, and a different profile of microbiota and metabolomics data were observed. Collectively, the data in the present study indicated that physical exercise in our model could be considered a mild stressor stimulus.

## Introduction

Intense exercise is known to exacerbate body stressors, such as oxidative stress and inflammation, the latter at both the muscular (1–3) and the gastrointestinal (GI) (4–6) levels. As a consequence, in performance sports, there is a high prevalence of GI problems both in humans, such as endurance runners (6–8), and in animals, such as horses (9, 10) or dogs (11). In a review paper by ter Steege et al. (12), several studies were cited that suggested that the key culprit behind GI symptoms during exercise was splanchnic hypoperfusion, which could lead to intestinal ischemia, thus subsequently damaging the intestinal epithelial cells and compromising the intestinal barrier function. Multiple studies involving humans have reported an exercise-induced increase in intestinal permeability (13). The tight junction (TJ) plays an important role in regulating the epithelial permeability by means of modifying the multiprotein complex [claudins and occludin (OCLN)] and/or promoting dysfunction to TJ regulatory proteins (i.e., zona-occludens) (14).

A downregulation of OCLN expression has been observed in different intestinal models, in which the permeability was strongly altered [i.e., inflammatory bowel disease (IBD), ulcerative colitis], and was downregulated (15, 16). Gut permeability is also influenced by protease-activated receptor-2 (PAR-2) expressed in the apical and basolateral membranes of intestinal epithelial cells (17). As described by a review (17), its activation induces an increase in permeability by means of impairment of the TJ functions, as shown in several epithelial and endothelial cell models (18–21). In different models including colitis and ischemia and reperfusion (I/R), PAR-2 transcription was upregulated in mouse, rodent, and horse models (21–23). Other markers of intestinal inflammation are calprotectin (CALP) and pro-inflammatory cytokines, which have been shown to be upregulated in IBD models (24–26).

Heme oxygenase-1 (HO-1) is an inducible cytoprotective stress-responsive protein induced by various stimuli, including oxidative stress I/R, heavy metals, and cytokines (27), the induction of which is usually associated with antioxidant, anti-apoptotic, and anti-inflammatory effects as reported by a review paper (28). In studies using murine experimental colitis models, HO-1 activity and expression were markedly increased, associated with the development of colitis, and the inhibition of HO activity potentiates colonic damage and inflammation (29, 30). Moreover, the relationship between physical exercise and increased HO-1 mRNA and protein expression/activity in different cells and tissues has already been demonstrated in rodents (31–34) as well as in humans (35, 36).

Cortisol is a well-known indicator of the stress response in the majority of mammals including dogs, with previous studies showing increased levels after exercise, such as agility work (37) and training in outdoor conditions (38, 39).

Many factors contribute to the maintenance of GI homeostasis. One of them is the secretion of immunoglobulin A (IgA), which coats the bacteria, favoring a tolerant, non-inflammatory relationship with the host (40) and the homeostatic control of the intestinal redox environment (41). Previous papers have reported that exercise may affect the levels of IgA in mice (42) and cause oxidative stress in dogs (43).

Emerging research has suggested that intense exercise could also affect the gut microbiota. In particular, cross-sectional studies have shown an overall increase in biodiversity with some compositional alterations, mainly in mucin degraders, lactate utilizers, and short-chain fatty acid (SCFA) producers, in the intestinal microbial ecosystem of professional athletes (44). Several factors are likely to be involved, including changes in diet, hydration levels and metabolic flux, altered gut motility, and also impaired gut barrier function, as a result of exercise-induced heat stress and ischemia (44). Given the fundamental role of the gut microbiota in maintaining host metabolic and immunological homeostasis (45), its monitoring during periods of intense physical activity could help to elucidate the mechanisms underlying the microbial response to exercise and understand if and how these are related to host performance.

The metabolome of fluids, which is made up of the ensemble of low-weight organic molecules, results from a complex interaction between endogenous and exogenous host factors, including the gut microbiota. As such, it has been shown to give important information regarding the overall effects of exercise in both humans and animals, with specific reference to the inflammatory status. The fecal metabolome seems to be no exception, at least in rats (46).

The exfoliated enterocytes contained in feces have recently been used as a tool to investigate the impact of therapies and nutritional regimens on GI functions (47, 48). In fact, stool is easy to obtain and has already been used in quantifying intestinal gene expression profiles from exfoliated epithelial cells in neonates (49, 50), as well as under pathological conditions to detect candidate molecular biomarkers (51–53). Exercise induces multiple biochemical changes, which may affect the gene expression of the transcripts involved in the mitochondrial metabolism in muscle (54) and oxidative stress, as assessed non-invasively (i.e., in saliva) in avalanche military dogs (55).

The aim of the present research was to evaluate the presence of a GI derangement in hunting dogs through a non-invasive sampling as a consequence of a period of intense exercise in comparison with samples collected at rest. To reach this goal, we selected a number of potential stress markers in fecal samples, including cortisol metabolites [fecal cortisol metabolite (FCM)], transcripts involved in epithelial integrity and inflammatory mechanisms [cytokines: interleukin (IL)-8, IL-6, IL-1β, and tumor necrosis factor alpha (TNFα); OCLN; CALP; PAR-2; and HO-1], IgA, and total antioxidant capacity (TAC) levels. Furthermore, we decided to profile the fecal metabolome, by means of high-resolution proton magnetic resonance spectroscopy (^1^H-NMR), and the microbiota, by 16S rRNA gene-based next-generation sequencing.

## Materials and Methods

### Experimental Design and Exercise

**Figure 1:**
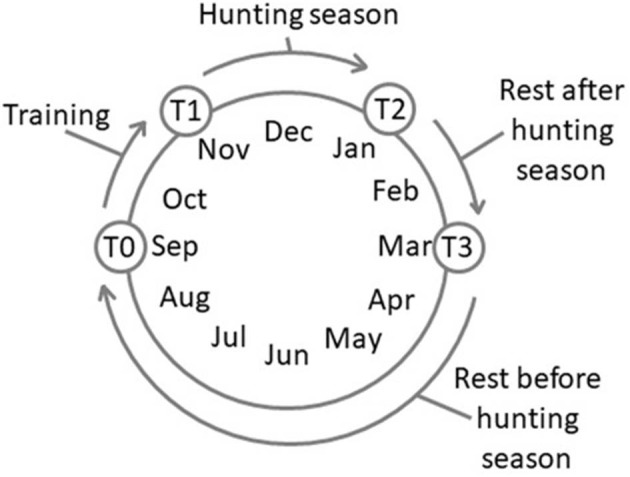
Schedule of the experimental time points. T0, rest before the training; T1, 60 days of training; T2, 60 days of hunting season; T3, 60 days of rest after hunting.

Four experimental time points were set: T0, after 180 days of complete rest (rest before the training session, September); T1, after 60 days of training, 3 days a week, 3 h each day (November); T2, after 60 days of wild boar hunting three times a week, 5–6 h each day (January); and T3, after 60 days of complete rest (rest after hunting season, March) (Figure 1). The physical activity carried out during both the training (T1) and the hunting (T2) periods was similar and consisted of a first phase of identifying and locating prey and a subsequent chase phase. The duration of these phases, due to the nature of the hunting itself, varied and was therefore impossible to standardize. All the dogs equally and simultaneously participated in each training/hunting session The training activity occurred on alternative days and was always conducted by the same person, the owner (not a professional trainer but an expert hunter fully aware of the goal of the research project), without any type of reinforcement.

### Animals

The exploratory study was carried out from September 2017 to March 2018 on nine hunting dogs. The dogs were of various ages (9.1 ± 5.0 years; mean ± SD), sexes (two unneutered males and seven spayed females), and breeds (three English Setter, three Segugio Italiano, two Dachsbacke, one Deutsch Kurzhaar) (Table 1). T0 body weight (BW) (19.3 ± 3.3 kg; mean ± SD) and the body condition score (BCS, calculated by using the 1–9 score proposed by Royal Canine SAS) are reported in Table 1. BW and BCS were also determined at each experimental point. The dogs, owned by a single owner, were housed in individual boxes and fed, once a day, with a commercial diet (Eko Adult, Russo Mangimi SpA, NA, Italy): crude protein 22%, crude fats and oils 9%, crude fiber 4.6%, and crude ash 11.2%. The food was administered in relation to the weight of the dog and to physical activity, increasing the dose by about 15% in T1 and T2 with respect to the rest periods. All the dogs underwent a physical examination by a veterinarian at the beginning of and during the trials. Only those who were clinically healthy were included in the study.

**Table 1:** Dogs included in the study.

**Dog**	**Breed**	**Gender**	**Age**	**BW (kg)**	**BCS 1–9**
1	Segugio Italiano	SF	4	18	6
2	Dachsbracke	M	14	20	6
3	Deutsch Kurzhaar	SF	4	25	5
4	English Setter	M	13	22	6
5	Segugio Italiano	SF	7	18	4
6	Segugio Italiano	SF	16	20	5
7	Dachsbracke	SF	2	13	5
8	English Setter	SF	12	18	5
9	English Setter	SF	10	20	6

*M, unneutered male; SF, spayed female; BW, body weight; BCS, body condition score at T0 (rest before training)*.

### Collection of the Fecal Sample

The samples were collected during the last week of each experimental period. In order to counterbalance the physiological fluctuations that occur within individuals, three samples for each time point were collected on different days. Specifically, at T1 and T2, the three samples were collected during the last week of physical activity on the day after the exercise session, while at T0 and T3, the three samples were collected on 3 consecutive days. The sample collection time was the same at each experimental point (after feeding in the late afternoon).

In agreement with the Italian law transposition of European Directive 2010/63 (DL 26/2014), the collection of fecal samples is not classified as a procedure, and it did not require any kind of authorization. This non-invasive sampling method was performed without any discomfort for the animals.

In total, 108 samples were collected: three for each dog at each of the four experimental times. The aforementioned three samples were pooled for the assays, leading to an overall sample number of 36 (one for each dog at each experimental time point).

Fresh fecal samples were collected by the owner within 1 h of defecation (late afternoon) and immediately stored at −20°C until analysis.

### RNA Extraction and Reverse Transcription

Lyophilized fecal samples (Modulyo EF4 1044, Edwards) were weighed and resuspended with Dulbecco's phosphate-buffered saline (DPBS) (w/v; 100 mg/ml) by vortex mixing (3 min). RNA extraction was performed using PureZol RNA isolation reagent (BioRad, Bio-RAD Laboratories Inc., California, USA) and a NucleoSpin RNA II kit (Macherey Nagel, Duren, Germany). Briefly, 1 ml of PureZol RNA isolation reagent was added to 100 μl of each sample and vortex mixed (3 min). Two hundred microliters of chloroform was then added to the suspension and mixed well. After incubation at room temperature (10 min), the samples were centrifuged (12,000 g for 10 min), and the aqueous phase was recovered. One volume of ethanol was added, and the resulting solution was loaded onto a NucleoSpin RNA Column (light blue ring) (NucleoSpin RNA II kit, Macherey Nagel). The RNA was then purified according to the manufacturer's instructions and spectrophotometrically quantified (A260 nm) (DeNovix Inc., Wilmington, DE, USA). RNA (1 μg) was then reverse-transcribed to cDNA using an iScript cDNA Synthesis Kit (Bio-RAD), arriving at a final volume of 20 μl. An additional sample of canine intestinal biopsy, collected from the duodenum of a dog with IBD (derived from a diagnostic procedure, performed at DIMEVET, with the express consent of the owner; endoscopy code 9290, March 19, 2018, sample code 14873), underwent RNA extraction, reverse transcription, and subsequent analysis (quantitative real-time PCR assay) as a positive control of inflammatory gene expression.

### Quantitative Real-Time PCR

Real-time quantitative PCR was carried out using a CFX 96 Real Time System (Bio-RAD) and SsoAdvanced™ Universal SYBR® Green Supermix (Bio-RAD). All the samples were analyzed in duplicate (10 μl/well), and the qPCR assays were carried out for different references [glyceraldehyde-3-phosphate dehydrogenase (GAPDH), TATA-box binding protein (TBP), tight junction protein 1 (TJP1), ribosomal protein L32 (RPL32), succinate dehydrogenase (SDHA), and interest genes (Il-8, IL-1β, IL-6, TNFα, OCLN, CALP, PAR-2, HO-1]. Primer sequences are reported in Table 2.

**Table 2:** List of primer pairs, amplicon size (bp), and accession number (AN) in the NCBI (National Center of Biotechnology Information) database.

**Gene**		**Primer sequence (5^**′**^->3^**′**^)**	**PCR (bp)**	**AN**	**References**
HO-1	F	GCCAGTGCCACGAAGTTC	164	NM_001194969	Present study
	R	TCCTCAGTGTCCTTGCTCAG			
CALP	F	ACCATGCTGACGGAACTGGAGAG	244	NM_001146144	Present study
	R	CCACGCCCACCTTTATCACCAATATG			
OCLN	F	CAGAGTCTTCCTATAAATCAAC	196	NM_001003195.1	Present study
	R	GTGTAGTCTGTCTCATAGTG			
PAR-2	F	TGAAGATCGCCTACCACATCCG	137	AB_458680	(56)
	R	CCAATACCGTTGCACACTGA			
IL-8	F	CTTCCAAGCTGGCTGTTGCTC	173	NM_001003200	(56)
	R	TGGGCCACTGTCAATCACTCTC			
IL-1β	F	GCTGCTGCCAAGACCTGAAC	112	XM_005630074	Present study
	R	GCTACAATGACTGACACGAAATGC			
TNFα	F	CCCAAGTGACAAGCCAGTAGCTC	146	NM_001003244	(56)
	R	ACAACCCATCTGACGGCACTATC			
IL-6	F	AAAGAGCAAGGTAAAGAATCAGGATG	126	NM_001003301	Present study
	R	CGCAGGATGAGGTGAATTGTTG			
GAPDH	F	TGTCCCCACCCCCAATGTATC	100	NM_001003142	(57)
	R	CTCCGATGCCTGCTTCACTACCTT			
TBP	F	CTATTTCTTGGTGTGCATGAG G	96	XM849432	(56)
	R	CCT CGG CATTCAGTCTTTTC			
TJP1	F	GCTGTGGAAGAAGATGAAGATG	175	NM_001003140	Present study
	R	CTCGGCAGACCTTGAAGTAG			
RPL32	F	GGCACCAGTCAGACCGATATG	209	NM_001252169	Present study
	R	GCACATCAGCAGCACTTCAAG			
SDHA	F	CGCATAAGAGCCAAGAAC	194	XM535807	Present study
	R	CCTTCCGTAATGAGACAAC			

*HO-1, heme oxygenase-1; CALP, calprotectin; OCLN, occludin; PAR-2, protease-activated receptor-2; IL, interleukin; TNFα, tumor necrosis factor alpha; GAPDH, glyceraldehyde-3-phosphate dehydrogenase; TBP, TATA-box binding protein; TJP1, tight junction protein 1; RPL32, ribosomal protein L32; SDHA, succinate dehydrogenase*.

Real-time efficiency was evaluated by amplification of a standardized amount of cDNA, starting from 150 ng with subsequent 5-fold dilutions (75, 15, 3, 0.6, and 0.12 ng), derived from both fecal sample–derived and intestinal cDNA (duodenal biopsy). The specificity of the amplified PCR products was verified by analysis of the melting curve and agarose gel electrophoresis. The relative gene expression was calculated as the fold increase using the 2^−ΔΔ*Ct*^ method (58) in relation to T0 (ΔΔCt = ΔCt _T1__or__T2__or__T3__group_ –ΔCt _T0_).

### Fecal Cortisol Metabolites Determination

Extraction from the feces was performed as previously described (59). Briefly, a methanol:water (v/v 4:1) solution was added to the feces in capped glass tube vials. The vials were then vortex mixed for 30 min using a multitube pulsing vortexer. Following centrifugation (1,500 g for 15 min), ethylic ether and NaHCO_3_ (5%) were added to 1 ml of supernatant. This preparation was then vortex mixed for 1 min on a multitube pulsing vortexer and centrifuged for 5 min (1,500 g). The ether portion was then separated and evaporated to dryness under an air-stream suction hood at 37°C; finally, the dry residue was dissolved into phosphate-buffered saline (PBS) 0.05 M, pH 7.5.

Radio immunological assay (RIA) was carried out according to Tamanini et al. (60). Analysis was carried out in duplicate. The parameters for analysis validation were: sensitivity 0.23 pg/mg; intra-assay variability 6.4%; inter-assay variability 9.7%; and specificity (%) of cortisol 100, corticosterone 9.5, 11α-hydroxy-progesterone 8.3, cortisone 5.3, 11α-deoxycortisol 5.0, progesterone 0.6, deoxycorticosterone 0.5, 20α-dihydrocortisone 0.4, testosterone 0.3, aldosterone 0.1, and dehydroepiandrosterone, 5α-pregnenolone, 17β-estradiol, and cholesterol <0.0001.

### Determination of Total IgAs and TAC

The IgA extraction was performed essentially as reported by Peters et al. (61).

Briefly, the lyophilized fecal samples were placed in 1 ml (w/v; 100 mg/ml) of extraction buffer (PBS containing 0.5% Tween 20 (Sigma-Aldrich, St. Louis, MO, USA) and a protease inhibitor cocktail (Sigma, 1 tablet/25 ml), and after the addition of three 3 mm glass beads, the samples were homogenized for 1 min with TissueLyser (50 Hz) (QIAGEN, Hilden, Germany). The homogenates were then centrifuged (1,500 g for 15 min), and the recovered supernatants were additionally centrifuged (15,000 g for 20 min). The supernatants were frozen at −20°C until analysis.

The IgA level was measured by a specific enzyme-linked immunosorbent assay (ELISA) kit (Dog IgA ELISA Quantitation Set, Bethyl Laboratories Inc., Montgomery, TX, USA). The analyses were carried out in duplicate. The parameters for analysis validation were: intra-assay variability 2.1% and inter-assay variability 12.8%. After checking the parallelism (*R*^2^ = 0.9849, unpublished data), we diluted the sample 1:75,000 and carried out the assay according to the manufacturer's instructions.

The TAC level was assayed by using an Antioxidant Assay Kit (item no. 709001; Cayman Chemical Company, Ann Arbor, MI, USA) according to the manufacturer's instructions and expressed as a Trolox equivalent.

### Metabolomics

The fecal samples were prepared for ^1^H-NMR analysis by vortex mixing for 5 min (80 mg of stool with 1 ml of deionized water). The mixtures were then centrifuged for 15 min at 18,630 g and 4°C. The supernatants (700 μl) were added to a D_2_O solution of 3-(trimethylsilyl)-propionic-2,2,3,3-d4 acid sodium salt (TSP) 10 mM and NaN_3_ 2 mM, set at pH 7.00 ± 0.02 with 1 M potassium phosphate buffer. Before analysis, the samples were centrifuged again at the above conditions.

The ^1^H-NMR spectra were recorded at 298 K using an AVANCE III spectrometer (Bruker, Milan, Italy) operating at a frequency of 600.13 MHz. In accord with Ventrella et al. (62), the signals from broad resonances originating from large molecules were suppressed by a Carr–Purcell–Meiboom–Gill (CPMG) filter composed by 400 echoes with a τ of 400 μs and a 180° pulse of 24 μs, for a total filter of 330 ms. The HOD residual signal was suppressed by means of pre-saturation. Each spectrum was acquired by summing up 256 transients using 32 K data points over a 7,184 Hz spectral window, with an acquisition time of 2.28 s. To apply NMR as a quantitative technique (63), the recycle delay was set to 5 s, taking into consideration the relaxation time of the protons under investigation. ^1^H-NMR spectra were baseline-adjusted by means of the peak detection according to the “rolling ball” principle (64) implemented in the baseline R package (65). A linear correction was then applied to each spectrum, so as to make the points pertaining to the baseline randomly spread around zero. Spectra have been horizontally aligned by employing the signal of TSP as a reference. The differences in water and fiber content among the samples were taken into consideration using probabilistic quotient normalization (66), applied to the entire spectra array.

The signals were assigned by comparing their chemical shift and multiplicity with the Human Metabolome Database (67) and Chenomx software data bank (Chenomx Inc., Canada, version 8.1).

### Microbial DNA Extraction and 16S rRNA Gene Sequencing

Microbial DNA was extracted from the fecal samples using the DNeasy Blood & Tissue kit (QIAGEN), with a modified protocol as previously described (68). Briefly, 250 mg of feces were resuspended in 1 ml of lysis buffer (500 mM NaCl, 50 mM Tris-HCl pH 8, 50 mM EDTA, 4% SDS). Four 3 mm glass beads and 0.5 g of 0.1 mm zirconia beads (BioSpec Products, Bartlesville, OK) were added to the fecal samples and homogenized with three bead-beating steps using the FastPrep instrument (MP Biomedicals, Irvine, CA) at 5.5 movements/s for 1 min, keeping the samples on ice for 5 min after each treatment. The samples were heated at 95°C for 15 min and centrifuged for 5 min at 13,000 g to pellet stool particles. The supernatants were collected, and 260 μl of 10 M ammonium acetate was added; the samples were then incubated on ice for 5 min and then centrifuged for 10 min at 13,000 g. One volume of isopropanol was added, and the supernatants were incubated on ice for 30 min. The nucleic acids were collected by centrifugation for 15 min at 13,000 g and washed with 70% ethanol. The pellets were then resuspended in 100 μl of Tris-EDTA (TE) buffer and treated with 2 μl of DNase-free RNase (10 mg/ml) for 15 min at 37°C. Protein removal and DNA purification using QIAamp Mini Spin columns (QIAGEN) were carried out according to the kit protocol. The DNA extracted was quantified using a NanoDrop ND-1000 spectrophotometer (NanoDrop Technologies, Wilmington, DE).

For each sample, the V3–V4 region of the 16S rRNA gene was sequenced as previously reported (69). Briefly, the DNA was amplified using the S-D-Bact-0341-b-S-17/S-D-Bact-0785-a-A-21 primers (70) with Illumina overhang adapter sequences. PCR products of ~460 bp were purified using a magnetic bead-based system (Agencourt AMPure XP; Beckman Coulter, Brea, CA), indexed by limited-cycle PCR using Nextera technology, and were additionally purified using Agencourt AMPure XP magnetic beads. Indexed libraries were pooled at an equimolar concentration, denatured, and diluted to 6 pmol/L before loading onto the MiSeq flow cell. Sequencing was carried out on an Illumina MiSeq platform using a 2 × 250 bp paired-end protocol, according to the manufacturer's instructions (Illumina, San Diego, CA). Sequencing reads were deposited in the National Center for Biotechnology Information Sequence Read Archive (NCBI SRA; BioProject ID PRJNA 589580).

### Bioinformatics and Statistical Analysis

Statistical analysis was carried out in R computational language (71). Differences among sampling points were assessed using the analysis of variance (ANOVA) test for repeated measures (*P*-value < 0.05 was considered statistically significant). Robust principal component analysis (rPCA) models were calculated as described by Hubert et al. (72), namely, by accepting an alpha value of 0.75. Differences in the mRNA data were evaluated using one-way ANOVA (*P*-value < 0.05 was considered statistically significant).

As for the gut microbiota analysis, raw sequences were processed using a pipeline combining PANDAseq (73) and QIIME 2 (74). High-quality reads were filtered and clustered into amplicon sequence variants (ASVs) at 99% similarity by means of an open-reference strategy carried out using dada2 (75). Taxonomy was assigned using the vsearch classifier (76) and the Greengenes database as a reference (release May 2013). Alpha rarefaction was carried out using Faith's phylogenetic index and the number of observed ASVs, while beta diversity was estimated by computing weighted and unweighted UniFrac distances. All the statistical analyses were carried out using R (version 3.1.3) and the packages vegan and made4. UniFrac distances were used for the principal coordinate analysis (PCoA), and the significance of data separation was tested using a permutation test with pseudo-*F* ratios (function adonis of vegan) and the ANOSIM test. The Wilcoxon test for paired data was used to assess significant differences in alpha diversity and taxon relative abundance between groups, while the Kruskal–Wallis test was used for multiple comparisons. A *P*-value < 0.05 was considered statistically significant.

## Results

### Animals

**Figure 2:**
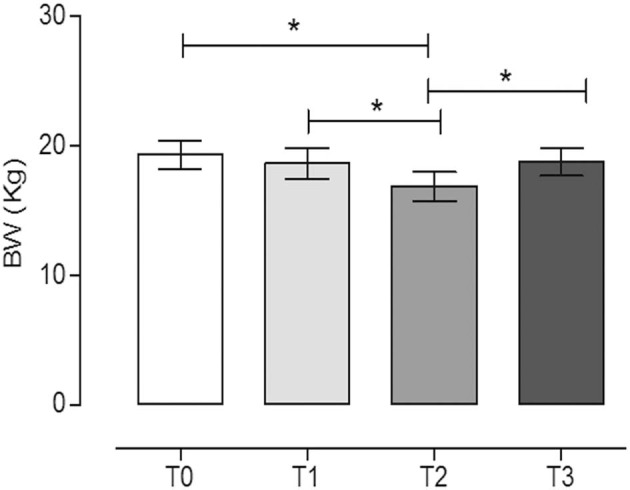
Body weight (BW) of dogs at the different time points. The physical activity induced a statistically significant decrease in BW after 60 days of hunting season (T2) (mean ± SEM) (*P* = 0.017). *Indicates *P* < 0.05 (repeated measures ANOVA, Tukey's multiple comparison test, *P* < 0.05).

In Figure 2, we report the variation in BW of the dogs during the trial.

The physical activity induced a statistically significant decrease of BW after 60 days of hunting season (T2, 16.9 ± 3.4) with respect to the rest periods (T0, 19.3 ± 3.3, and T3, 18.8 ± 3.2) (*P* = 0.017, repeated measures ANOVA, Tukey's multiple comparison test, *P* < 0.05). On the contrary, the training period did not significantly influence the BW (T1, 18.7 ± 3.6) (repeated measures ANOVA, Tukey's multiple comparison test, *P* < 0.05). The percentages of BW reduction at T1, T2, and T3 with respect to T0 were 3.2, 12.7, and 2.9%, respectively.

The BCSs of the dogs recorded during the trial were (median, min–max): T0 (4, 5, 5, 6); T1 (4, 5, 5, 6); T2 (3, 4, 4); and T3 (4, 5, 5, 6). Similarly to BW, only T2 (60 days after hunting season) was statistically different from rest periods (T0 and T3) and the period after 60 days of training (T1) (repeated measures ANOVA, Friedman test, Dunn's multiple comparison test, *P* < 0.05).

### Real-Time Quantitative Reverse Transcription PCR for PAR-2, HO1, CALP, OCLN, IL-8, IL-1β, IL-6, and TNFα

**Figure 3:**
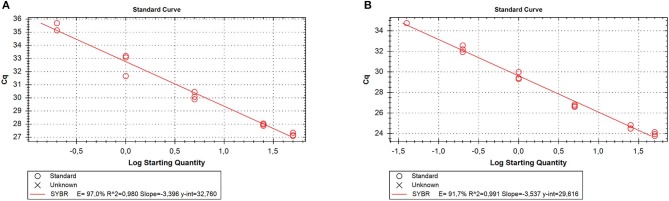
Quantitative real time PCR (qRT-PCR) efficiency for the reference gene [glyceraldehyde-3-phosphate dehydrogenase (GAPDH)]. Five different 5-fold dilutions of the stool **(A)** or tissue **(B)** samples were assayed. Cq, cycle quantification; E, efficiency.

RNA was extracted from all the samples with a yield of 336.35 ± 147.8 ng/10 mg dry feces. Of the reference genes analyzed, only GAPDH was always detectable; therefore, it was used as a reliable internal reference for qPCR normalization. To evaluate the matrix effect, we determined qPCR efficiency for GAPDH in the stool and tissue samples. The results showed that the efficiency was similar in both samples (97 and 91.7%, respectively) (Figure 3), indicating that RNA isolated from feces did not contain particular PCR inhibitors.

The presence and specificity of the PCR products were verified using melting curve analysis and agarose gel electrophoresis. The transcripts of GAPDH, HO-1, CALP, OCLN, and PAR-2 were detectable in the majority of the samples analyzed (GAPDH 33/36, HO-1 29/36, PAR-2 27/36, CALP 21/36, OCLN 26/36), although with a huge variability regarding the range of gene expression both between the dogs and regarding the time points.

**Figure 4:**
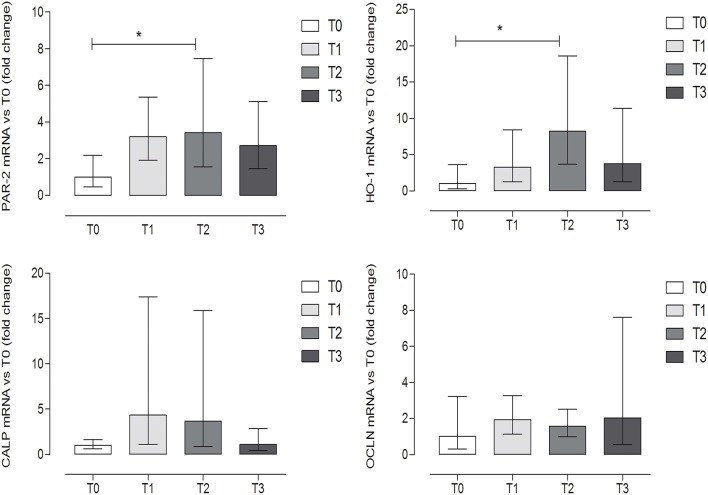
Gene expression of protease-activated receptor-2 (PAR-2), heme oxygenase-1 (HO-1), calprotectin (CALP), and occludin (OCLN) evaluated by qRT-PCR, at the different time points. Relative gene expression of PAR-2, HO-1, CALP, and OCLN in the fecal samples of dogs at rest before training (T0), after 60 days of training (T1), after 60 days of hunting season (T2), and at 60 days of rest after hunting (T3). The mRNA data are expressed as fold change with respect to T0. *Indicates *P* < 0.05 (one-way ANOVA, *P* < 0.05, *post-hoc* Tukey's test). *P*-values: *P* = 0.042 for PAR-2; *P* = 0.028 for HO-1; *P* = 0.1704 for CALP; *P* = 0.6338 for OCLN. Error bars represent the range of gene expression.

The expression levels of OCLN and CALP did not show significant differences among groups (*P* = 0.6338 and *P* = 0.1704, respectively) (one-way ANOVA, Tukey's multiple comparison test, *P* < 0.05, Figure 4). On the contrary, a statistically significant increase was observed at T2 (after 60 days of hunting season) for PAR-2 and HO-1 as compared to T0 (*P* = 0.042 and *P* = 0.028, respectively) (one-way ANOVA, Tukey's multiple comparison test, *P* < 0.05, Figure 4). Very low or undetectable expression levels were observed for the genes encoding the cytokines (IL-8, IL-1β, IL-6, TNFα) (very low 7/36, undetectable 29/36) and for the other reference genes (TPB, TJP1, RPL32, SDHA) (very low 8/36, undetectable level 28/36).

### FCM Determination

**Figure 5:**
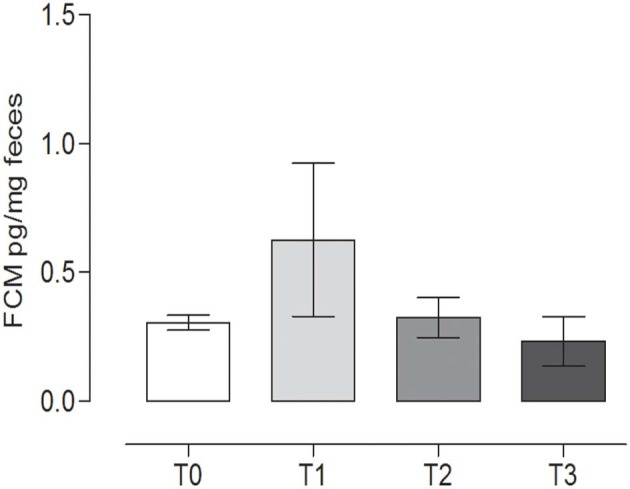
Fecal cortisol metabolites (FCMs) at the different time points. The concentration of cortisol metabolites (mean ± SEM) in the fecal samples of the dogs at rest before training (T0), after 60 days of training (T1), after 60 days of hunting season (T2), and at 60 days of rest after hunting (T3). No statistically significant differences (*P* = 0.2760) were observed (repeated measures ANOVA, Tukey's multiple comparison test, *P* < 0.05).

No statistically significant differences were observed in FCM content during the trial (*P* = 0.270) (repeated measures ANOVA, *P* < 0.05). The concentration of FCMs at T0 was 0.31 ± 0.03 pg/mg feces, while at T1, the level was 0.63 ± 0.29 pg/mg feces (Figure 5).

### Determination of Total IgA in Stools

**Figure 6:**
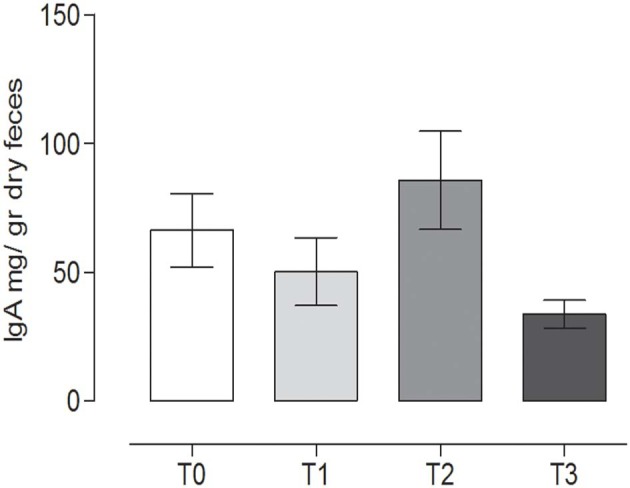
Immunoglobulin A (IgA) concentrations in the stool at the different time points. The IgA concentrations (mean ± SEM) in the fecal samples of dogs at rest before training (T0), after 60 days of training (T1), after 60 days of hunting season (T2), and at 60 days of rest after hunting (T3). No statistically significant differences (*P* = 0.065) were observed (repeated measures ANOVA, Tukey's multiple comparison test, *P* < 0.05).

The IgA content in the canine fecal samples at the different time points is reported in Figure 6. No statistically significant differences among the groups were observed (*P* = 0.065) (repeated measures ANOVA, *P* < 0.05).

### Determination of Total Antioxidant Activity

**Figure 7:**
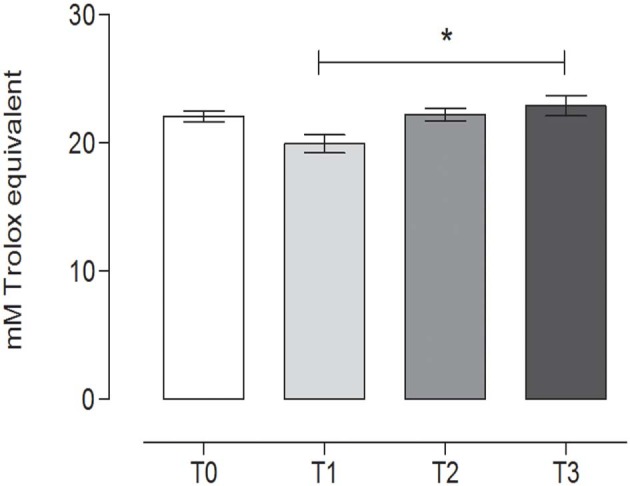
Total antioxidant capacity (TAC) in the fecal samples at the different time points. The TAC value (mean ± SEM) in the fecal sample at rest before training (T0, 22.74 ± 0.46), after 60 days of training (T1, 19.82 ± 0.79), after 60 days of hunting season (T2, 22.16 ± 0.56), and at 60 days of rest after hunting (T3, 22.89 ± 0.89). The TAC was significantly lower at T1 than at T3 (*P* = 0.0213). *Indicates *P* < 0.05 (repeated measures ANOVA, Tukey's multiple comparison test, *P* < 0.05).

TAC showed a slight variation during the study, with a statistically significant difference between T1 (19.82 ± 0.79, mean ± SD) (after 60 days of training) and the rest after the hunting season, T3 (22.89 ± 0.89, mean ± SD) (60 days of rest after hunting) (*P* = 0.0213) (repeated measures ANOVA, *P* < 0.05, Figure 7).

### Metabolomics of the Feces

In order to explore the changes in the fecal metabolome of the dogs involved in the study, the ^1^H-NMR spectra were registered. Seventy-three molecules could be quantified. Seventeen molecules, reported in Table 3, showed a concentration that differed among the time points investigated.

**Table 3:** Temporal dynamics of the fecal metabolome of hunting dogs following physical activity.

	**T0**	**T1**	**T2**	**T3**	***P*-value**
Formate	4.95 × 10^−5^ ± 1.47 × 10^−4^ ^b^	2.32 × 10^−4^ ± 5.96 × 10^−5^ ^a^	2.37 × 10^−4^ ± 4.25 × 10^−5^ ^a^	2.82 × 10^−4^ ± 1.76 × 10^−4^ ^a^	6.72E-04
Uridine	1.85 × 10^−4^ ± 8.83 × 10^−5^ ^b^	3.65 × 10^−4^ ± 1.14 × 10^−4^ ^a^	3.89 × 10^−4^ ± 1.30 × 10^−4^ ^a^	3.98 × 10^−4^ ± 1.71 × 10^−4^ ^a^	3.89E-04
3-Hydroxyphenylacetate	2.63 × 10^−4^ ± 4.25 × 10^−4^ ^b^	1.69 × 10^−3^ ± 9.38 × 10^−4^ ^a^	1.71 × 10^−3^ ± 8.45 × 10^−4^ ^a^	1.26 × 10^−3^ ± 7.45 × 10^−4^ ^ab^	1.13E-05
Galactose	3.02 × 10^−5^ ± 9.33 × 10^−5^ ^c^	5.16 × 10^−4^ ± 3.48 × 10^−4^ ^b^	2.78 × 10^−4^ ± 1.07 × 10^−4^ ^a^	3.39 × 10^−4^ ± 2.84 × 10^−4^ ^ab^	4.16E-06
Arabinose	8.57 × 10^−4^ ± 4.92 × 10^−4^ ^b^	2.99 × 10^−3^ ± 1.16 × 10^−3^ ^a^	2.50 × 10^−3^ ± 8.47 × 10^−4^ ^a^	2.05 × 10^−3^ ± 1.11 × 10^−3^ ^a^	1.13E-05
Fucose	4.52 × 10^−5^ ± 2.02 × 10^−4^ ^b^	5.88 × 10^−4^ ± 2.30 × 10^−4^ ^a^	4.72 × 10^−4^ ± 3.19 × 10^−4^ ^a^	4.36 × 10^−4^ ± 1.89 × 10^−4^ ^a^	6.88E-05
1,3-Dihydroxyacetone	1.23 × 10^−5^ ± 2.44 × 10^−5^ ^b^	1.39 × 10^−4^ ± 1.24 × 10^−4^ ^a^	9.51 × 10^−5^ ± 7.63 × 10^−5^ ^a^	9.42 × 10^−5^ ± 8.98 × 10^−5^ ^a^	3.35E-04
Galacturonate	6.51 × 10^−5^ ± 9.02 × 10^−5^ ^c^	1.94 × 10^−4^ ± 8.84 × 10^−5^ ^b^	1.08 × 10^−4^ ± 6.96 × 10^−5^ ^abc^	1.35 × 10^−4^ ± 5.67 × 10^−5^ ^a^	1.30E-05
Malate	7.92 × 10^−4^ ± 6.07 × 10^−4^ ^b^	1.83 × 10^−3^ ± 9.29 × 10^−4^ ^a^	1.46 × 10^−3^ ± 1.01 × 10^−3^ ^ab^	2.32 × 10^−3^ ± 1.93 × 10^−3^ ^a^	3.48E-02
Threonine	8.07 × 10^−4^ ± 5.81 × 10^−4^ ^b^	2.14 × 10^−3^ ± 6.66 × 10^−4^ ^a^	1.91 × 10^−3^ ± 3.42 × 10^−4^ ^a^	1.99 × 10^−3^ ± 5.16 × 10^−4^ ^a^	1.17E-03
Glycine	2.07 × 10^−3^ ± 4.68 × 10^−4^ ^b^	4.82 × 10^−3^ ± 4.85 × 10^−3^ ^ab^	2.80 × 10^−3^ ± 7.04 × 10^−4^ ^ab^	3.33 × 10^−3^ ± 9.62 × 10^−4^ ^a^	1.70E-03
Methanol	2.37 × 10^−4^ ± 2.06 × 10^−4^ ^b^	6.02 × 10^−4^ ± 2.77 × 10^−4^ ^a^	4.48 × 10^−4^ ± 1.29 × 10^−4^ ^ab^	4.98 × 10^−4^ ± 2.82 × 10^−4^ ^ab^	2.02E-02
Proline	2.39 × 10^−4^ ± 1.31 × 10^−4^ ^b^	6.64 × 10^−4^ ± 1.49 × 10^−4^ ^a^	6.58 × 10^−4^ ± 3.27 × 10^−4^ ^ab^	6.57 × 10^−4^ ± 1.36 × 10^−4^ ^a^	2.10E-05
Trimethylamine (TMA)	3.99 × 10^−4^ ± 2.36 × 10^−4^ ^ab^	2.65 × 10^−4^ ± 1.44 × 10^−4^ ^b^	4.04 × 10^−4^ ± 1.27 × 10^−4^ ^a^	4.44 × 10^−4^ ± 3.00 × 10^−4^ ^ab^	4.53E-02
Homocystine	3.17 × 10^−4^ ± 5.05 × 10^−4^ ^b^	2.42 × 10^−3^ ± 1.56 × 10^−3^ ^a^	2.17 × 10^−3^ ± 1.41 × 10^−3^ ^a^	1.52 × 10^−3^ ± 8.90 × 10^−4^ ^a^	6.01E-05
Methylamine	1.84 × 10^−4^ ± 8.64 × 10^−5^ ^b^	4.20 × 10^−4^ ± 2.34 × 10^−4^ ^ab^	3.81 × 10^−4^ ± 1.08 × 10^−4^ ^a^	3.02 × 10^−4^ ± 1.21 × 10^−4^ ^b^	1.13E-03
Valine	1.80 × 10^−3^ ± 7.53 × 10^−4^ ^b^	2.51 × 10^−3^ ± 8.29 × 10^−4^ ^ab^	2.66 × 10^−3^ ± 5.34 × 10^−4^ ^ab^	2.85 × 10^−3^ ± 9.57 × 10^−4^ ^a^	4.42E-02

*Concentration (mmol/g, mean ± SD) of the molecules significantly differed among groups (Repeated Measure ANOVA, P < 0.05)*.

*For each molecule, different superscript letters identify significant differences among the groups (P < 0.05). For each molecule P value was reported.

**Figure 8:**
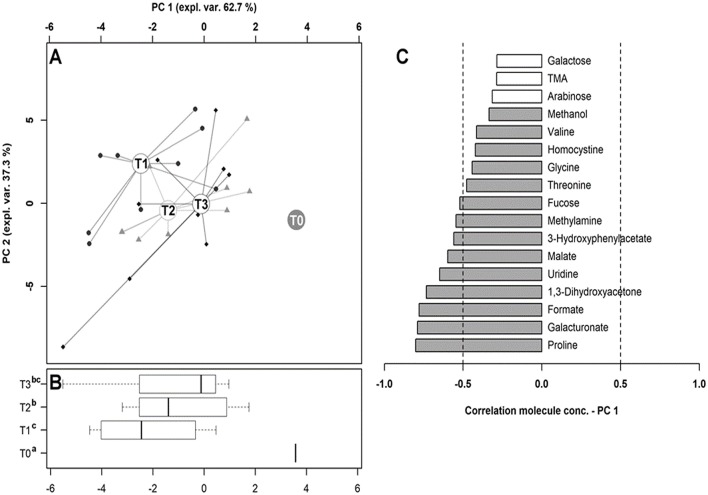
Diversity of the fecal metabolome in hunting dogs. The robust principal component analysis (rPCA) model built on the space constituted by the molecules listed in Table 3, the concentration of which at T0 was subtracted from the other time points. In the score plot **(A)**, the empty circles highlight the medians of the samples collected at each time point. The position of the samples along PC1 is summarized in the box plots in **(B)**, where the different superscript letters identify the significant differences among the groups (repeated measures ANOVA, *P* < 0.05). The loading plot **(C)** reports the correlation between the concentration of each substance and its importance over PC1. The significant correlations (repeated measures ANOVA, *P* < 0.05) are highlighted with gray bars.

To observe the overall trends driving the changes that these molecules underwent, their concentrations were used as a basis for an rPCA model, as depicted in Figure 8. Along PC1 of its score plot (Figure 8A), representing as much as 62.7% of the entire sample's variability explained by the PCA, the metabolomes of the dogs at T0 and T1 were characterized by the highest and the lowest scores, respectively, while the fecal metabolomes of the dogs at T2 and T3 appeared in intermediate positions. Specifically, the samples at T0, T1, and T2 appeared to be significantly separated from one other, while the metabolome at T3 was not distinguishable from that at T1 or T2. Figure 8C is a pictorial representation that highlights how all the molecules that have changed significantly over time tended to have the lowest concentrations at T0. The molecules mainly responsible for grouping the samples in this respect were proline, galacturonate and formate, 1,3-dihydroxyacetone, uridine, malate, 3-hydroxyphenylacetate, methylamine, and fucose.

### The Structure and the Variations of the Gut Microbiota of Hunting Dogs as Related to Physical Activity

The 16S rRNA gene-based next-generation sequencing yielded a total of 1,390,231 high-quality reads, with an average of 39,720 ± 12,005 sequences per sample, binned in 1,460 ASVs at 99% similarity.

**Figure 9:**
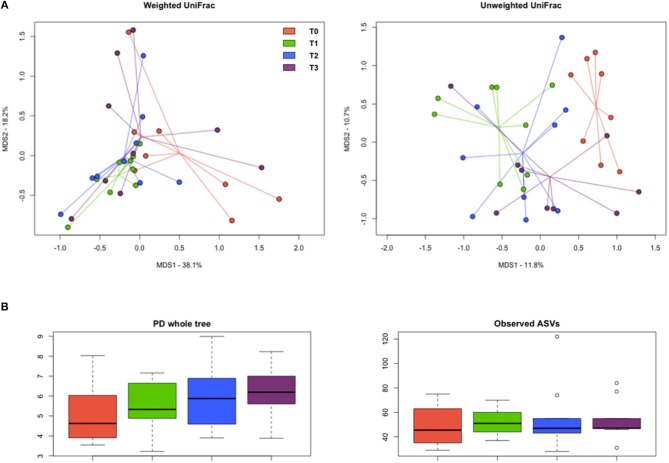
Diversity of the gut microbiome in hunting dogs. **(A)** Principal coordinate analysis (PCoA) plots showing the beta diversity of the gut microbial communities of the study groups (rest before training, T0; after 60 days of training, T1; after 60 days of hunting season, T2; at 60 days of rest after hunting, T3), based on weighted and unweighted UniFrac distances. A significant separation among groups was found (*P* < 0.03, permutation test with pseudo-*F* ratios; *P* ≤ 0.02, ANOSIM). **(B)** Box plots showing alpha diversity, computed with Faith's phylogenetic index (PD whole tree) and the number of observed amplicon sequence variants (ASVs).

The PCoA of inter-sample variation based on weighted and unweighted UniFrac distances showed significant separation among the study groups (*P* < 0.03, permutation test with pseudo-*F* ratios; *P* ≤ 0.02, ANOSIM) (Figure 9A). In particular, according to both the adonis and the ANOSIM statistics applied to the unweighted UniFrac-based ordination, the samples at T1 and T2 segregated from those at T0 (*P* < 0.005), while the T3 samples occupied an intermediate position (Table S1). No significant differences were found in alpha diversity, even though Faith's phylogenetic index showed an increasing trend over time (Figure 9B).

**Figure 10:**
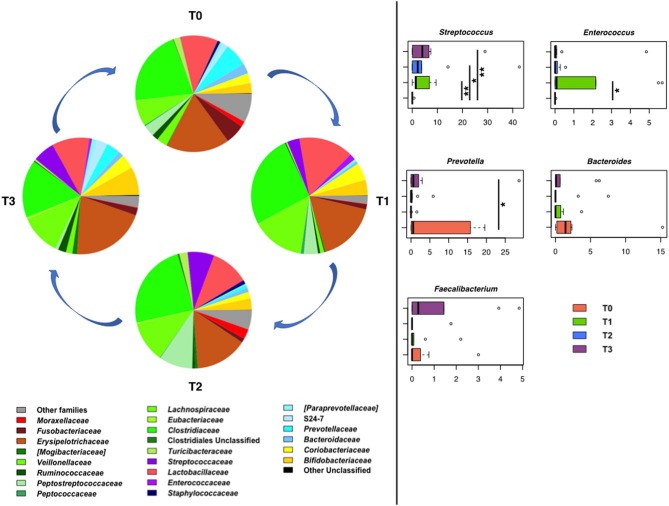
Temporal dynamics of the gut microbiome in hunting dogs following physical activity. **Left**, pie charts representing the average values of family-level relative abundances at each time point (rest before training, T0; after 60 days of training, T1; after 60 days of hunting season, T2; at 60 days of rest after hunting, T3). **Right**, box plots showing the distribution of the relative abundances of significantly enriched or depleted bacterial genera over time. *P < 0.04; **P < 0.001 (Wilcoxon test). For *Bacteroides* and *Faecalibacterium*, only a decreasing trend was observed.

In line with the literature available regarding the gut microbiota of healthy dogs (77, 78), the fecal microbial profiles at the baseline were dominated by the phylum Firmicutes (relative abundance, mean ± SEM, 69.6 ± 8.1%), with Bacteroidetes (12.0 ± 5.2%), Actinobacteria (6.7 ± 3.1%), Proteobacteria (6.0 ± 2.4%), and Fusobacteria (5.5 ± 4.1%) as minor components. Similar proportions were observed during training, hunting, and the subsequent rest period, except for a reduction in the relative abundance of Proteobacteria after training (*P* < 0.01, Wilcoxon test). *Clostridiaceae, Erysipelotrichaceae*, and *Lactobacillaceae* were the major families of the baseline microbiota (relative abundance > 10%). Following training, an increase in the relative abundance of *Streptococcaceae* and *Enterococcaceae* was observed (*P* < 0.05). Such an increase persisted for *Streptococcaceae* (*P* = 0.008) until the rest period after hunting, while for *Enterococcaceae*, relative abundance values comparable to the baseline were restored (Figure 10). In contrast, diminished proportions were observed for *Prevotellaceae* and *Ruminococcaceae* after training (*P* < 0.04).

Consistent with the above results, the main discriminant genera were *Streptococcus* and *Enterococcus*, the relative abundance of which was significantly greater at T1 than at T0 (*P* < 0.04), and *Prevotella*, the proportions of which decreased after training (*P* ≤ 0.03) (Figure 10).

Although not significant, a decreasing trend was observed for *Faecalibacterium* and *Bacteroides* after physical activity (i.e., training and hunting) compared to both rest periods (i.e., before the training and after the hunting season). At rest, the baseline relative abundance of *Enterococcus* was restored, whereas the proportions of *Streptococcus* remained higher than the baseline (*P* = 0.008).

## Discussion

Inx this study, we evaluated the fluctuations of different stress markers in fecal samples of hunting dogs during physical activity and at rest. The main limitation for such studies lies within the difficulty in standardizing the training protocol (wild boar hunting) and the management of privately owned animals (diet, housing, treatments, etc.). In order to try and overcome this problem, we chose a group of dogs owned by the same person, in this case, one of the animal technicians of the Veterinary Department. He is indeed routinely involved in the husbandry and care of animals for both clinical and experimental purposes, and he was fully aware of the goals of the experiment and of the potential biases imputable to variations in the management of animals enrolled in such trials.

This choice has added a limiting factor to the study, which, being exploratory, included a low number of dogs of different breeds and ages, variables known to potentially influence the results (79); nonetheless, the study design allowed for a high level of standardization in terms of dogs' management, making for reliable results despite the relatively low sample size.

Typically, performance dogs are kept at 4–5/9 BCS due to the great chance of body condition loss during endurance activity, and the diet was calculated to support this condition. Weight loss is known to be related with some of the parameters measured in this research, for instance, microbiota (80), metabolomics profile (81), and cortisol (82). The expected weight loss observed during the trial has to be interpreted as a direct consequence of physical activity and not of a caloric restriction, so its potential effects on the measured parameters could be considered as a direct consequence of the physical activity.

In the model used in the present study, fecal cortisol, a well-known marker of stress in dogs, did not show any difference across the time points. In previous investigations, increases in cortisol concentration after sustained exercise had been observed in horses and humans, while the data regarding dogs were contradictory. In fact, some papers have reported increased levels of cortisol (83, 84), while others agreed with the present study in reporting no significant changes (85, 86). In particular, Pastore et al. (37) and Ando et al. (39) reported that cortisol increased right after exercise but returned to baseline levels shortly after, suggesting a mild transient stress. Similarly, in the present study, cortisol showed a transient non-significant increase during the first phase of activity (60 days of training, T1) only. Moreover, all samples were collected during a short-day period (autumn–winter), avoiding the reported interference of photoperiod on the cortisol concentrations (87).

Intestinal IgA secretion is considered to be an important indicator of mucosal immunity. Similar to cortisol, the literature regarding the effect of exercise on IgA secretion is contradictory, reporting either an increase or a decrease in intestinal IgA in mice (42, 88, 89). Based on the present data, training might not influence IgA concentration, confirming that exercise does not drastically alter canine intestinal immune homeostasis.

A previous paper indicated an increase in oxidative stress in hunting dogs after exercise (43): in accordance with this paper, the data in the present study also showed a significant and transient reduction in TAC during T1 (60 days of training), in relation to T3 (60 days of rest after hunting), suggesting an increase in oxidative stress following the resumption of physical activity.

In agreement with the human results, in the present study, we were able to detect different biomarkers' transcripts in dog stool samples. Among the studied genes, PAR-2 and HO-1 were significantly altered after the hunting period. To date, the relationship between exercise and increased HO-1 expression has been well-documented in different tissue and animal models (31–34). Such an increase is likely to restore HO-1 protein expression levels after 60 days of training (T1), when oxidative stress is high, as confirmed by the TAC data. As for PAR-2, it is well-described in intestinal models of I/R injury that the receptor is strongly activated by the tryptase released, for the most part by the mast cell infiltrate, with a consequent increase in paracellular permeability by means of the activation of myosin light chain kinase (MLCK) and myosin phosphatase (MP) (17, 90); once activated, the receptor is translocated to the lysosomes and degraded (23, 91). In different animal models regarding intestinal I/R injury, an increase in the PAR-2 transcript has been observed (21–23), consistent with the present data showing a slight but significant increase in PAR-2 mRNA levels at the end of the hunting period (T2). This similar trend in different models may be due to the fact that during exercise, the blood flow is diverted from the gut to the periphery, creating an I/R-like scenario (92) with the potential consequent activation of PAR-2. It has been reported that PAR-2 activation may directly affect cytoskeleton contraction by triggering the phosphorylation of MLCK with subsequent changes in TJ permeability, as demonstrated in *in vitro* epithelial models (19, 20). However, the unchanged expression level of OCLN suggests that the PAR-2 receptor activation in our model is insufficient to induce damage at the TJ level, and so we were unable to predict the impairment of barrier permeability.

The lack of the detection of cytokine transcripts and the absence of changes in CALP mRNA levels additionally reinforced the authors' assumptions, i.e., that physical exercise in the present model could be considered mild and did not result in a strong inflammatory GI response.

Nevertheless, metabolomics data indicate that some intestinal disorder occurred. A two-step approach regarding the metabolome of the feces, based on univariate/multivariate analyses, allowed hypothesizing the overall trends that the fecal molecule profiles underwent as a consequence of resting, training, and hunting. The samples collected at T2, T3, and T0 showed median scores along PC1 of −1.39, −0.12, and 3.69, respectively. From a metabolomic perspective, therefore, the recovery of baseline conditions seemed to be linearly related to time. The metabolomes of the dogs at rest before the training (T0) were markedly different from all the other time points. The greatest modifications from this long period of rest were associated with training, while the subsequent activities seemed to lead to a progressive return of the metabolome to the baseline characteristics. This confirmed a metabolic shift between rest and activity. Of the molecules leading to such a circular trend, some, as expected, pertained to the biochemical processes connected to energy (46). This was the case for malate, which is part of the TCA cycle. Interestingly, of the sugars, glucose showed no significant differences, while fucose and galacturonate did. Of the molecules that were, for the most part, modified in the present study, 1,3-dihydroxyacetone, formate, and uridine should be mentioned. In a previous experiment (93), these three molecules were found to be altered in mouse feces after the administration of probiotics, probably as a result of the modification of the intestinal microbiota. In particular, the increase in 1,3-dihydroxyacetone, an intermediate in fructose metabolism, was found to lead to an increase in intestinal permeability, which is a known consequence of prolonged strenuous exercise in both dogs (94) and humans (7).

Consistent with the abovementioned assumptions, the gut microbiota structure also underwent a rearrangement during training and tended to approach the initial configuration in the rest period following the hunt. In line with the literature available regarding exercise and gut microbiota, this rearrangement was characterized by: (1) a tendency toward increased biodiversity (95); (2) decreased relative abundance of widely prevalent commensals (i.e., *Prevotella* and *Ruminococcaceae* members) (96–98); and (3) increased proportions of subdominant taxa, including *Streptococcus, Enterococcus*, and *Slackia* (96, 99). The majority of these changes were transient, which additionally reinforced the hypothesis of a reversible non-drastic alteration of the intestinal ecosystem. However, this was not true for *Streptococcus*, which, similarly to *Enterococcus*, includes species known to act as pathobionts, i.e., capable of pathogenic expansion under unfavorable conditions, compromising and eventually translocating across the epithelial barrier, with potentially severe implications for the host health (100). It is also worth noting that *Streptococcus* spp. are capable of proteolytically interacting with PARs (101) and have previously been positively correlated with uridine levels, probably by means of the activity of cytidine deaminase (102), which suggests a major role for this bacterial genus in exercise response. On the other hand, negative correlations have so far been found between uridine as well as DHA and *Bacteroides* (103), the relative abundance of which tended to be gradually reduced over the course of activity and no longer restored. Although transient and non-significant, the depletion of *Faecalibacterium*, a well-known butyrate producer with multiple health-promoting activities (104), constitutes another red flag for possible GI (and systemic) complications and should be monitored in cases of intense and prolonged physical activity.

## Conclusion

The aim of the present explorative study was to evaluate the presence of a GI derangement in hunting dogs through a non-invasive sampling as a consequence of a period of intense exercise in comparison with samples collected at rest.

We evaluated a number of potential stress markers in canine fecal samples. In particular, FCMs, IgA levels, and the TAC were measured. Moreover, the expression of selected genes was investigated, and microbiota and metabolomics analyses were carried out. Exercise induced a variation in gene expression, a reduction in TAC, and a modulation of the microbiome and metabolome profiles. Despite the intense physical activity required for hunting wild boar, the animals did not seem to show signs of particularly high stress under conditions of programmed training; all the data were consistent with a limited degree of alteration of intestinal homeostasis. Despite the limited statistical power of the study related to the relatively low number of subjects enrolled, the present findings are encouraging for the development of a non-invasive monitoring method for detecting the effect of exercise in dogs using a multidisciplinary integrated approach.

## Data Availability Statement

The microbiota dataset generated for this study can be found in the NCBI SRA (BioProject ID PRJNA 589580).

## Author Contributions

MF and PB conceived the project. MP and AG participated in the inclusion and clinical examination of the dogs, and performed the diagnostic endoscopy. AZ carried out the gene expression and IgA and TAC analyses. LL and CZ were responsible for the metabolomics investigation. MB and ST were responsible for the gut microbiota analysis. PA carried out the FCM analysis. AZ, LL, ST, and MB carried out the statistical analyses and wrote the original draft. MF and PB reviewed and edited the draft. All authors read and approved the final version of the manuscript.

## Conflict of Interest

The authors declare that the research was conducted in the absence of any commercial or financial relationships that could be construed as a potential conflict of interest.
